# Evaluation of characteristics and prognosis of COVID-19 patients requiring invasive mechanical ventilation during dominance of nonvariant, alpha, delta, and omicron variants in tertiary hospitals of Japan

**DOI:** 10.1186/s12879-024-09131-4

**Published:** 2024-02-20

**Authors:** Kazuhito Sakuramoto, Daiki Wada, Shuhei Maruyama, Takashi Muroya, Fukuki Saito, Yasushi Nakamori, Yasuyuki Kuwagata

**Affiliations:** 1 Department of Emergency and Critical Care Medicine, Kansai Medical University Hospital, 2-3-1 Shinmachi, Hirakata, Osaka, 573-1191 Japan; 2 Department of Emergency and Critical Care Medicine, Kansai Medical University General Medical Center, 10-15 Fumizono-cho, Moriguchi, Osaka, 570-8507 Japan

**Keywords:** COVID-19, Invasive mechanical ventilation, SARS-CoV-2, Omicron variant

## Abstract

**Background:**

In November 2021, the B.1.1.529 (omicron) variant of severe acute respiratory syndrome coronavirus 2 (SARS-CoV-2) was detected in South Africa and subsequently rapidly spread around the world. Despite the reduced severity of the omicron variants, many patients become severely ill after infection and undergo invasive mechanical ventilation, but there are few reports on their background and prognosis throughout all variant periods. This study aimed to evaluate risk factors affecting patients requiring invasive mechanical ventilation with each variant of COVID-19 pandemic in Japan from nonvariants to omicron variants.

**Method:**

This retrospective observational study was conducted at the Department of Emergency and Critical Care Medicine, Kansai Medical University Hospital and Kansai Medical University Medical Center, Osaka, Japan, from March 2020 to March 2023. Eligible patients were those who underwent invasive ventilation for COVID-19 pneumonia. We set the primary endpoint as in-hospital mortality. Multivariable logistic regression analysis adjusted for clinically important variables was performed to evaluate the clinical outcomes.

**Results:**

We included 377 patients: 118 in the Nonvariant group, 154 in the Alpha group, 42 in the Delta group, and 63 patients in the Omicron group. Mortality rates for each group were 23.7% for the Nonvariant group, 12.3% for the Alpha group, 7.1% for the Delta group, and 30.5% for the Omicron group. Patient age was significantly associated with increased mortality (adjusted odds ratio [AOR]: 1.097; 95% confidence interval [CI]: 1.057–0.138, *P* < 0.001). Immunodeficiency (AOR: 3.388, 95% CI: 1.377–8.333, *P* = 0.008), initial SOFA score (AOR: 1.190, 95% CI: 1.056–1.341, *P* = 0.004), dialysis prior to COVID-19 (AOR: 3.695, 95% CI: 1.117–11.663, *P* = 0.026), and smoking history (AOR: 2.548, 95% CI: 1.153–5.628, *P* = 0.021) were significantly associated with increased mortality. Differences in variants were not significant factors associated with high mortality.

**Conclusion:**

We compared the background and prognosis of patients with COVID-19 pneumonia requiring invasive mechanical ventilation between SARS-CoV-2 variants. In these patients, differences in variants did not affect prognosis. Hospital mortality in critically ill COVID-19 patients was significantly higher in the older patients with bacterial coinfection, or patients with immunodeficiency, COPD, and chronic renal failure on dialysis.

**Supplementary Information:**

The online version contains supplementary material available at 10.1186/s12879-024-09131-4.

## Background

The omicron variant of SARS-COV-2 was first reported in South Africa in November 2022 and has since spread worldwide [1]. Omicron’s virulence and ability to escape from neutralizing antibodies are more potent than those of the alpha and delta variants, but the rates of severe illness and mortality are reported to be low [2–4]. During the epidemic of the omicron variant, the decrease in severity of COVID-19 may be affected by the increased immunity due to vaccination and the lower ability of the omicron variant to migrate to the lungs. Despite this reduced severity, some patients become severely ill after infection with omicron variants and must undergo invasive mechanical ventilation (IMV), but there are few reports on the background and prognosis of these patients. In COVID-19 pandemic, we experienced waves I–III due to nonvariants, wave IV due to B.1.1.7 (alpha variant), wave V due to B.1.617.2 (delta variant), and waves (VI–VIII) due to B.1.1.529 (omicron variant) in Japan. In this study we compared the patient background and prognosis of COVID-19 patients who received mechanical ventilation in each period during the pandemic. Throughout all periods, risk factors affecting the prognosis of COVID-19 patients requiring IMV were also analysed and evaluated.

## Methods

### Study design and participants

**Fig. 1:**
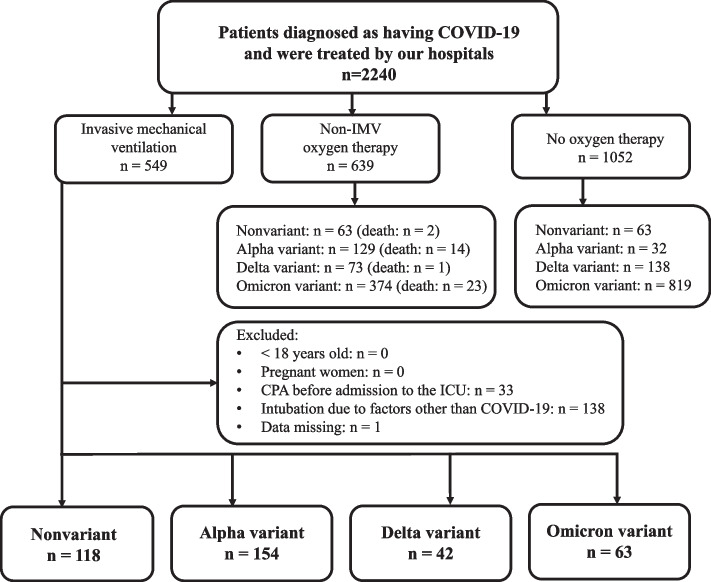
Flow of the patients with COVID-19 in our hospitals. CPA: cardiopulmonary arrest, ICU: intensive care unit, IMV: invasive mechanical ventilation

**Fig. 2:**
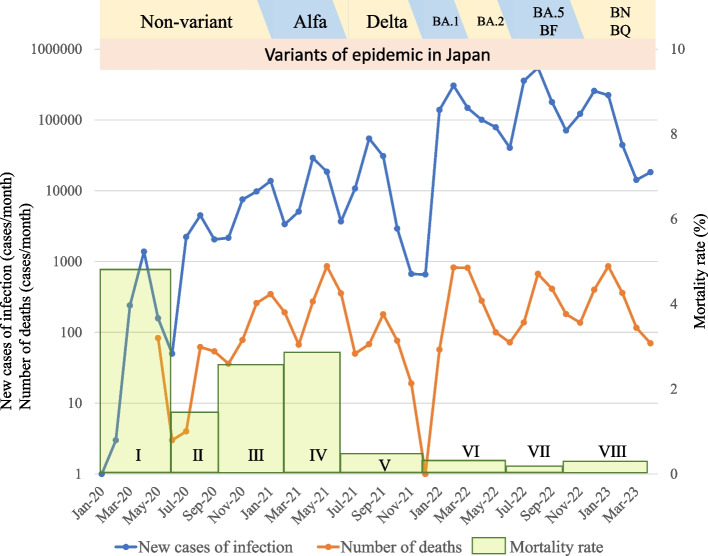
Epidemic curve of SARS-CoV-2 in Osaka, Japan

This was a retrospective observational study conducted at the Department of Emergency and Critical Care Medicine, Kansai Medical University Hospital and Kansai Medical University Medical Center, Osaka, Japan, from March 2020 to March 2023. Eligible patients were those with SARS-CoV-2 detected by polymerase chain reaction (PCR) testing of sputum or nasopharyngeal swabs and COVID-19-specific pneumonia on chest computed tomography (CT) scan who underwent IMV. The CT images were interpreted by an emergency physician and a radiologist with reference to the Expert Consensus of the Radiological Society of North America (RSNA). Atypical appearances in COVID-19, such as isolated consolidation without ground-glass opacities, were excluded [5]. Patients aged < 18 years, pregnant women, patients in cardiopulmonary arrest before admission to the intensive care unit (ICU), and patients intubated due to factors other than COVID-19 pneumonia (bacterial pneumonia, septic shock without pneumonia, or perioperative management) were excluded. Participants were classified into four groups according to when they were infected with SARS-CoV-2 as follows: March 2020 to February 2021, the Nonvariant group; March 2021 to June 2021, the Alpha group; July 2021 to December 2021, the Delta group; and January 2022 and thereafter, the Omicron group (Fig. 1). The epidemic curve of the number of new SARS-CoV-2 infections, deaths, and mortality rates in Osaka Prefecture, where Kansai Medical University is located, which provides the background for this study, is shown in Fig. 2 [6].

### Treatment protocol for COVID-19

With regard to antivirals, prior to the approval of remdesivir, patients were treated with favipiravir for 14 days. After approval of remdesivir, remdesivir was administered for 5–10 days in general, although molnupiravir was administered for 5 days in some patients with poor renal function. Patients who were unvaccinated or seronegative despite vaccination were given anti-SARS-CoV-2 monoclonal antibodies at the decision of their physician. Corticosteroids were administered from the first case in March 2020. Methylprednisolone 125 mg for 3 days, followed by 40 mg/day. The dose of methylprednisolone was reduced or terminated after confirming peak serum KL-6 and LDH [7]. Patients who were treated with anti-cytokine agents received tocilizumab until baricitinib was approved in April 2021. After its approval, baricitinib was administered except in patients with poor renal function.

### Data collection

The data we collected included background factors such as age, sex, BMI, vaccination history, smoking history, date of SARS-CoV-2 infection and days from infection to admission, days from infection to intubation, comorbidities, immunodeficiency, blood test results at admission and peak, positive blood culture, PaO_2_/FIO_2_ (PF) ratio after intubation, ventilator free days (number of days from day 1 to day 28 that the ventilator was not used), treatment for COVID-19, use of anti-MRSA drugs and broad-spectrum antibiotics, presence of barotrauma, tracheostomy, in-hospital mortality, and causes of death. Fully vaccinated was defined as having received two or more vaccinations. Immunodeficiency was defined as patients who met any of the following criteria: taking any immunosuppressive drugs for collagen disease, organ transplantation, or other reasons; receiving chemotherapy for malignant diseases of solid organs; or having a history of chemotherapy for hematologic malignant diseases.

### Statistical analysis and ethical concerns

Categorical data are summarized as frequencies and proportion, and continuous variables are shown as the median and interquartile 25–75th percentile range (IQR). Fisher’s exact test was used for group comparisons of categorical variables, with an asterisk (*) indicating a significant difference between the group and the Omicron group on multiple comparisons adjusted with Bonferroni’s method. Kruskal-Wallis tests were used for group comparisons of continuous variables, with an asterisk (*) indicating a significant difference between the group and the Omicron group on the Dunn-Bonferroni post-hoc test. Next, a logistic regression model was used to calculate the odds ratio (OR) with confidence interval (CI) for the risk of hospital death of a patient with COVID-19 pneumonia requiring IMV for each parameter. In addition, multivariate logistic regression was used to control for factors that may be confounded by age, BMI, smoking history, vaccination, SARS-CoV-2 variants, dialysis prior to COVID-19, immunodeficiency, Charlson Comorbidity Index, initial Sequential Organ Failure Assessment (SOFA) score, PF ratio after intubation, and initial values of C-reactive protein (CRP), procalcitonin (PCT), and lactate dehydrogenase (LDH). A *P* value of < 0.05 was considered to indicate statistical significance. Statistical analysis was performed with SPSS 28.0 software (IBM Corp, USA) and the EZR software program, version 1.54 [8].

This study was conducted according to the principles expressed in the Declaration of Helsinki and approved by the Institutional Review Board of Kansai Medical University Medical Center (study number 2023099), which waived the requirement for written informed consent due to the retrospective study design.

## Results

### Characteristics of participating patients

In total, 2240 consecutive patients were diagnosed as having COVID-19 and were admitted to our tertiary hospitals. Of these, 377 patients met the entry criteria for this study (Fig. 1). There were 118 patients in the Nonvariant group, 154 in the Alpha group, 42 in the Delta group, and 63 in the Omicron group.

The median age was significantly higher in the Omicron group, at 75 (IQR 67.5–81.5) years. The rate of fully vaccinated patients was 0% in the Nonvariant and Alpha groups but was 49% in the Omicron group. The Omicron group was significantly more immunocompromised (36.5%) and had a significantly higher Charlson Comorbidity Index of 4 (IQR 1–8). Initial blood tests showed elevated levels of CRP (15.3 mg/dl; IQR 8.8–21.3), PCT (0.36 ng/ml; IQR 0.13–0.89), and D-dimer (2.7 μg/ml; IQR 1.3–7.1) in the Omicron group, which are affected by complications of bacterial infection. Values of LDH and KL-6, which are elevated in COVID-19 pneumonia, were not significantly different in each group.

For treatment of COVID-19, corticosteroids were administered in all patients, whereas the use of antivirals, neutralizing antibodies, and anti-cytokines was significantly different in each group due to changing recommendations in the guidelines (Table 1).

**Table 1:** Patient background, laboratory data, and intervention by variant type of SARS-CoV-2

	Nonvariant *n* = 118	Alpha *n* = 154	Delta *n* = 42	Omicron *n* = 63	*P*-value
Patient background					
Age, yrs., (range)	72.5 (61–79)	67 (56–74)^a^	55.5 (46–69)^a^	75 (67.5–81.5)	< 0.001
Male gender, n (%)	84 (71)	117 (76)	29 (69)	44 (70)	0.659
Smoking history, n (%)	64 (63)	90 (65)	21 (51)	31 (61)	0.459
Fully vaccinated, n (%)	0^a^	0^a^	3 (7)^a^	31 (49)	< 0.001
BMI (range)	23.3 (21.0–25.8)^a^	24.6 (22.4–27.6)	25.3 (20.8–29.0)	24.7 (20.9–29.5)	0.076
Immunodeficiency, n (%)	13 (11.0)^a^	9 (5.8)^a^	1 (2.4)^a^	23 (36.5)	< 0.001
Dialysis prior to COVID-19 infection, n (%)	7 (5.9)	15 (9.7)	0	2 (3.2)	0.081
Charlson Comorbidity Index (range)	1 (0–2)^a^	1 (0–2)^a^	1 (0–2)^a^	4 (1–6)	< 0.001
Days from onset to admission (range)	7 (4–9)^a^	7.5 (5–10)^a^	6 (4–7)	4 (1–8)	< 0.001
Days from onset to intubation (range)	8 (6–10)^a^	8 (6–11)^a^	6 (5–8)	5 (2–8)	< 0.001
SOFA on ICU admission (range)	6 (5–8)	7 (4–8)	6 (4–8)	7 (4.5–8)	0.605
PF ratio after intubation (range)	258 (187–340)^a^	219 (153–303)^a^	248 (180–322)^a^	141 (97–233)	< 0.001
Initial laboratory data					
CRP, mg/dl, (range)	9.5 (6.0–16.3)^a^	8.8 (4.4–14.2)^a^	9.7 (5.1–20.1)	15.3 (8.8–21.3)	< 0.001
Procalcitonin, ng/ml, (range)	0.19 (0.11–0.43)	0.17 (0.08–0.36)^a^	0.24 (0.13–0.95)	0.36 (0.13–0.89)	0.002
D-dimer, μg/ml, (range)	1.6 (0.8–4.3)	1.2 (0.5–3.7)^a^	1.0 (0.5–1.7)^a^	2.7 (1.3–7.1)	< 0.001
LDH, U/L, (range)	404 (309–509)	436 (361–554)	516 (428–626)	404 (315–529)	0.002
KL-6, U/ml, (range)	389 (266–587)	413 (266–713)	346 (242–606)	445 (290–787)	0.489
Intervention for COVID-19					
Antivirals	116 (98.3)	150 (97.4)	42 (100)	60 (93.8)	0.228
Remdesivir, n (%)	72 (61.0)^a^	115 (74.7)^a^	40 (95.2)	58 (92.1)	< 0.001
Favipiravir, n (%)	58 (49.2)^a^	41 (26.6)^a^	2 (4.8)	0	< 0.001
Molnupiravir, n (%)	0^a^	0^a^	0^a^	9 (14.3)	< 0.001
Neutralizing antibody, n (%)	0^a^	0^a^	2 (4.8)	13 (20.6)	< 0.001
Methylprednisolone, n (%)	118 (100)	154 (100)	42 (100)	63 (100)	1
Anti-cytokine drugs	97 (82.2)	148 (96.1)^a^	41 (97.6)^a^	42 (66.7)	< 0.001
Tocilizumab, n (%)	97 (82.2)^a^	103 (66.9)^a^	4 (9.5)	5 (7.9)	< 0.001
Baricitinib, n (%)	0^a^	48 (31.2)^a^	39 (92.9)^a^	37 (58.7)	< 0.001
Antibacterial					
Anti-MRSA antibacterial, n (%)	63 (53.4)	96 (62.3)	26 (61.9)	44 (69.8)	0.172
Broad-spectrum antibacterial, n (%)	85 (72.0)	124 (80.5)	25 (59.5)	47 (74.6)	0.0422

^a^Significant difference between the group and the Omicron group on post-hoc test. *BMI* body mass index, *SOFA* Sequential Organ Failure Assessment, *ICU* intensive care unit, *PF ratio* PaO_2_/FiO_2_ ratio, *CRP* C-reactive protein, *LDH* lactate dehydrogenase, *KL-6* sialylated carbohydrate antigen KL-6, *MRSA* methicillin-resistant *Staphylococcus aureus*, Broad-spectrum antibacterial: carbapenem or tazobactam/piperacillin

Among the 2240 consecutive patients, 109 deaths occurred in total. Mortality rates for each group were 23.7% for the Nonvariant group, 12.3% for the Alpha group, 7.1% for the Delta group, and 30.5% for the Omicron group. The Omicron group had a significantly higher mortality rate than that of the Alpha group. Sixty (87%) deaths were due to COVID-19 pneumonia (Table 2).

**Table 2:** Prognostic factors by mutation type of SARS-CoV-2

Prognostic factor	Nonvariant *n* = 118	Alpha *n* = 154	Delta *n* = 42	Omicron *n* = 63
Peak LDH, U/L, (range)	536 (415–751)	529 (435–673)	538 (458–761)	544 (408–793)
Peak KL-6, U/ml, (range)	809 (514–1410)	795 (485–1359)	505 (386–823)	730 (502–1299)
Peak *β-D Glucan ≥ 20 pg/ml, n (%)*	24 (20.3)	34 (22.1)	5 (11.9)	18 (28.6)
Positive blood culture, n (%)	20 (16.9)^a^	21 (13.6)^a^	2 (4.8)^a^	34 (54.0)
Ventilator-free days, days, (range)	17 (0–21)	17 (0–21)	16 (4–19)	0 (0–19)
Barotrauma, n (%)	13 (11.0)	18 (11.7)	0	4 (6.3)
Tracheostomy, n (%)	23 (19.5)	41 (26.6)	10 (23.8)	23 (36.5)
In-hospital mortality, n (%)	28 (23.7)	19 (12.3)^a^	3 (7.1)	19 (30.5)
Causes of death				
Covid-19 pneumonia, n	26	18	2	14
Others, n	Bacterial pneumonia, 1 Lung cancer, 1	Heart failure, 1	Bacterial sepsis, 1	Bacterial sepsis, 2 Bacterial pneumonia, 1 Duodenal bleeding, 1 Intestinal necrosis, 1

^a^Significant difference between the group and the Omicron group on post-hoc test. *LDH* lactate dehydrogenase, *KL-6* sialylated carbohydrate antigen KL-6

### Risk factors for mortality in patients with COVID-19 pneumonia requiring IMV

The Alpha group was associated with a lower risk of hospital mortality versus the Omicron group, with an OR of 0.326 (95% CI: 0.158–0.670, *P* = 0.002). The Delta group was also associated with a lower risk of hospital mortality versus the Omicron group, with an OR of 0.178 (95% CI: 0.049–0.648, *P* = 0.009) (Table 3).

**Table 3:** Univariate and multivariate analysis of risk factors for hospital death

	Univariate analysis			Multivariate analysis		
	*P* value	Odds ratio	95% Confidence interval	*P* value	Odds ratio	95% Confidence interval
Age	< 0.001	1.087	1.057–1.118	< 0.001	1.097	1.057–1.138
Gender, male	0.731	0.904	0.507–1.611			
Fully vaccinated	0.411	0.703	0.304–1.627			
BMI	0.010	0.923	0.868–0.981			
Smoking history	0.138	1.593	0.861–2.947	0.021	2.548	1.153–5.628
Charlson Comorbidity Index	< 0.001	1.230	1.097–1.379			
Immunodeficiency	< 0.001	4.427	2.295–8.541	0.008	3.388	1.377–8.333
Dialysis prior to COVID-19 infection	0.016	2.930	1.225–7.006	0.026	3.695	1.117–11.663
Variants (nonvariant vs omicron)	0.880	0.720	0.363–1.429			
Variants (alpha vs omicron)	0.002	0.326	0.158–0.670			
Variants (delta vs omicron)	0.009	0.178	0.049–0.648			
PF ratio after intubation	0.102	0.998	0.995–1.000			
Initial SOFA	< 0.001	1.209	1.103–1.324	0.004	1.190	1.056–1.341
Initial CRP	0.083	0.970	0.937–1.004			
Initial procalcitonin	0.219	1.023	0.987–1.061			
Initial LDH	0.362	0.999	0.998–1.001			

*BMI* body mass index, *PF ratio* PaO_2_/FiO_2_ ratio, *SOFA* Sequential Organ Failure Assessment, *CRP* C-reactive protein, *LDH* lactate dehydrogenase

Multivariable logistic regression model for hospital death (age, BMI, smoking history, vaccination, variants, dialysis before COVID-19, immunodeficiency, Charlson Comorbidity Index, initial SOFA, PF ratio after intubation, initial CRP, initial procalcitonin, initial LDH)

After multivariable analyses were performed, significant factors associated with high mortality were age (adjusted OR: 1.097; 95% CI: 1.057–0.138, *P* < 0.001), immunodeficiency (adjusted OR: 3.388, 95% CI: 1.377–8.333, *P* = 0.008), initial SOFA score (adjusted OR: 1.190, 95% CI: 1.056–1.341, *P* = 0.004), dialysis prior to COVID-19 (adjusted OR: 3.695, 95% CI: 1.117–11.663, *P* = 0.026), and smoking history (adjusted OR: 2.548, 95% CI: 1.153–5.628, *P* = 0.021) (Table 3).

## Discussion

In this study, we compared the background and prognosis of patients with COVID-19 pneumonia requiring IMV during four viral epidemic waves in Japan (nonvariant, alpha variant, delta variant, and omicron variant). While many similar reports have included patients in whom SARS-CoV-2 was detected and who received IMV, we included patients in whom SARS-CoV-2 was detected who received IMV due only to COVID-19 pneumonia. We excluded patients who did not have COVID-19-specific pneumonia on chest CT at the time of admission. Patients with lung abnormalities on chest CT were excluded if they were diagnosed as having bacterial pneumonia, aspiration pneumonia, atelectasis, heart failure, or other causes. Since the outbreak of the omicron variant, there has been an increase in the number of patients brought to the emergency department for causes other than COVID-19 and who tested positive for SARS-CoV-2 by PCR testing prior to admission.

In addition to differences in the characteristics of each variant, factors such as the healthcare system, available drugs, and the prevalence of vaccines against SAR-CoV-2 may have influenced the comparison of patient backgrounds. Patients in the Omicron group were significantly older than those in the Alpha and Delta groups. According to data from Osaka Prefecture, in which our hospital is located, among the patients positive for SARS-CoV-2 during the omicron variant epidemic, the number of patients aged 70 years or older was 235,060 (9.0%). In comparison, 8368 (15.1%) older patients were in the alpha variant epidemic, and 4131 (4.1%) were in the delta variant epidemic [9]. One possible reason for the significant difference in the age of the groups is the large difference in the number of older patients who tested positive for SARS-COV-2 in those periods.

As the drugs used against COVID-19, corticosteroids were the treatment of choice for COVID-19 in all patients even before the guidelines recommended them [10], whereas significant differences existed between the groups for other agents administered. Among the antiviral drugs, only favipiravir, which was being investigated in Japan, could be used in the early stages of the epidemic [11]. Subsequently, after remdesivir was approved, its use increased [12]. During the omicron variant epidemic, molnupiravir was given to patients with poor renal function. As a result, one of these antivirals was administered to most patients. We had been using tocilizumab since the beginning of the epidemic [13] and then switched to baricitinib when it was approved [14]. After tocilizumab was also approved, we chose between the two depending on renal function [15]. No anti-cytokine drugs were administered in cases diagnosed as complicated by bacterial pneumonia, based on the findings of the initial CT and blood tests. In the omicron variant, the rate of anti-cytokine drugs was low (66.7%) because many of the patients were older and had bacterial pneumonia from the admission. These data showed that despite differences in the drugs, each variant group was treated similarly for COVID-19.

Initial blood test results showed that CRP, PCT, and D-dimer were significantly higher in the Omicron group. As KL-6 and LDH, which have been reported to be elevated in severe COVID-19 pneumonia [7, 16], are comparable, the elevations in CRP, PCT, and D-dimer are likely due to bacterial coinfection. Patton et al. reported that bacterial co-infection is a major risk factor for mortality, regardless of SARS-CoV-2 variants [17]. The detailed data in their report indicate that the rate of bacterial coinfection was 4.7% for the alpha variant, 5.4% for the delta variant, and 10.7% for the omicron variant. In the cases of death in our cohort, the positivity rate of βD-glucan was 49% and that of blood culture was 37.7%. The positivity rate for βD-glucan was not significantly different for each variant, although the Omicron variant had a significantly higher rate of positive blood cultures. One of the factors for the high mortality in patients with the omicron variant was likely to have been bacterial or fungal co-infection. Within the limitation of the retrospective design of this study, we could show COVID 19 pneumonia-related mortality.

In the present study, in-hospital mortality was highest in the Omicron group at 30.5%. Univariate analysis showed that the omicron variant had a significantly higher risk of death than the alpha and delta variants. A report from Brazil compared patients admitted to the ICU with nonvariant, gamma/delta variants, and omicron variants and reported that prognosis was better with omicron variants, but there was no difference in prognosis when limited to patients who required invasive ventilation [18]. A report from France showed that ICU mortality in COVID-19 patients admitted to the ICU was lower for the omicron variant than for the delta variant, but there was no difference in ICU mortality when restricted to patients admitted due to pneumonia [4]. Our study is also consistent with these studies in that the SARS-CoV-2 variant did not remain as a risk for in-hospital mortality of patients who required IMV for COVID-19 pneumonia in the multivariate analysis. The SARS-CoV-2 variant was also not a significant risk factor in a multivariate logistic regression restricting the primary endpoint to COVID-19 associated mortality (Supplementary Table 1). Importantly, our multivariate analysis of the risk of in-hospital mortality for all patients over the entire period showed age, immunodeficiency, smoking history, dialysis prior to COVID-19 infection, and high SOFA score at admission to be the significant risk factors. In the present study, the reason for more in-hospital deaths in the Omicron group may be that it included more patients who were older, immunocompromised, or had complicated bacterial infections with elevated CRP or PCT. In other words, even with omicron variant infections, which are said to have reduced rates of severe illness and mortality [2, 3], patients who are older, immunocompromised, have chronic obstructive pulmonary disease (COPD) due to smoking, or chronic renal failure on dialysis may require careful treatment and follow-up.

This study has several limitations. The major limitation is the lack of sufficient sample sizes and the use of retrospective data analysis. In addition, other factors, such as the degree of urgency in the health care system and differences in population age groups and health care supply by country may also affect prognosis. Since the emergence of the omicron variant, the number of elderly people with bacterial pneumonia following SARS-COV-2 infection has increased. In this study, patients whose main CT findings were consolidation or atelectasis possibly bacterial pneumonia were excluded. Because COVID-19 pneumonia also causes sepsis and it can be combined with bacterial coinfection, these exclusion criteria may possibly be inadequate. Further studies are needed to accurately determine risk factors affecting the prognosis of COVID-19 patients requiring IMV.

## Conclusions

We compared the patient background and prognosis of patients with critical COVID-19 pneumonia between the SARS-CoV-2 variants. In patients with severe COVID-19 pneumonia requiring invasive ventilation, differences in the variants did not affect prognosis. Hospital mortality in critically ill COVID-19 patients was significantly higher in the older patients with bacterial coinfection, or patients with immunodeficiency, COPD, and chronic renal failure on dialysis.

### Supplementary Information

**Supplementary Material 1.**

## Data Availability

The datasets used and analysed during the current study are available from the corresponding author on reasonable request.

## Abbreviations

BMI: Body mass index
COPD: Chronic obstructive pulmonary disease
COVID-19: Coronavirus disease 2019
CRP: C-reactive protein
CT: Computed tomography
ICU: Intensive care unit
KL-6: Sialylated carbohydrate antigen KL-6
LDH: Lactate dehydrogenase
PCR: Polymerase chain reaction
PCT: Procalcitonin
PF ratio: PaO_2_/FIO_2_ ratio
SOFA: Sequential Organ Failure Assessment
SARS-CoV-2: Severe acute respiratory syndrome coronavirus 2
