# Supplementary material for: Evaluation of characteristics and prognosis of COVID-19 patients requiring invasive mechanical ventilation during dominance of nonvariant, alpha, delta, and omicron variants in tertiary hospitals of Japan
Source: BMC Infect Dis. 2024 Feb 20;24:223. doi: 10.1186/s12879-024-09131-4 (PMC10877910; doi:10.1186/s12879-024-09131-4)
Supplement: Supplementary file 1 — Supplementary Material 1. [file 12879_2024_9131_MOESM1_ESM.docx]

| **Supplementary table 1** Univariate and multivariate analysis of risk factors for COVID-19 associated death | | | | | | |
| --- | --- | --- | --- | --- | --- | --- |
|  | **Univariate analysis** | | | **Multivariate analysis** | | |
|  | **P value** | **Odds ratio** | **95% Confidence interval** | **P value** | **Odds ratio** | **95% Confidence interval** |
| Age | <0.001 | 1.088 | 1.056-1.121 | <0.001 | 1.095 | 1.055-1.138 |
| Gender, male | 0.612 | 0.855 | 0.467-1.566 |  |  |  |
| Fully vaccinated | 0.772 | 0.872 | 0.345-2.206 |  |  |  |
| BMI | 0.044 | 0.937 | 0.880-0.998 |  |  |  |
| Smoking history | 0.143 | 1.620 | 0.849-3.091 |  |  |  |
| Charlson Comorbidity Index | 0.085 | 1.114 | 0.985-1.260 |  |  |  |
| Immunodeficiency | <0.001 | 3.479 | 1.754-6.899 | 0.020 | 3.019 | 1.193-7.642 |
| Dialysis prior to COVID-19 infection | 0.020 | 2.894 | 1.179-7.106 |  |  |  |
| Variants (nonvariant vs omicron) | 0.977 | 0.989 | 0.474-2.066 |  |  |  |
| Variants (alpha vs omicron) | 0.050 | 0.463 | 0.214-1.001 |  |  |  |
| Variants (delta vs omicron) | 0.026 | 0.175 | 0.038-0.816 |  |  |  |
| PF ratio after intubation | 0.102 | 0.998 | 0.995-1.000 |  |  |  |
| Initial SOFA | <0.001 | 1.199 | 1.090-1.318 | <0.001 | 1.238 | 1.101-1.392 |
| Initial CRP | 0.152 | 0.974 | 0.939-1.010 | 0.036 | 0.951 | 0.908-0.997 |
| Initial procalcitonin | 0.366 | 1.018 | 0.980-1.058 |  |  |  |
| Initial LDH | 0.297 | 0.999 | 0.998-1.001 |  |  |  |
| BMI: body mass index, PF ratio: PaO_2_/FiO_2_ ratio, SOFA: Sequential Organ Failure Assessment, CRP: C-reactive protein, LDH: lactate dehydrogenase  Multivariable logistic regression model for hospital death (age, BMI, smoking history, vaccination, variants, dialysis before COVID-19, immunodeficiency, Charlson Comorbidity Index, initial SOFA, PF ratio after intubation, initial CRP, initial procalcitonin, initial LDH) | | | | | | |
